# Guided Limited Maxillectomy and Staged Septal–Palatal Reconstruction for Low-Grade Chondrosarcoma of the Hard Palate: A Case Report and Literature Review

**DOI:** 10.3390/jcm15051722

**Published:** 2026-02-25

**Authors:** Kito franck, Thibaut Van Zele, Matthias Ureel, Renaat Coopman, Benjamin Denoiseux

**Affiliations:** 1 Department of Oral & Maxillofacial Surgery, Ghent University Hospital, Corneel Heymanslaan 10, 9000 Ghent, Belgium; matthias.ureel@uzgent.be (M.U.); renaat.coopman@ugent.be (R.C.); benjamin.denoiseux@uzgent.be (B.D.); 2 Department of Otorhinolaryngology, Ghent University Hospital, Corneel Heymanslaan 10, 9000 Ghent, Belgium

**Keywords:** low-grade chondrosarcoma, hard palate, maxillary tumor, image-guided limited maxillectomy, 3D cutting guide, posterior pedicle lateral nasal wall flap, palatal rotation flap, staged septal–palatal reconstruction, case report

## Abstract

Chondrosarcoma of the maxillofacial skeleton is a rare malignant tumor characterized by cartilaginous differentiation and locally invasive growth. Diagnosis is particularly challenging in low-grade tumors because histological features often overlap with those of benign chondroma. We describe a 62-year-old woman with a recurrent cartilaginous tumor of the hard palate. After previous resections in 2013 and 2022, a third recurrence was detected. MRI showed a lobulated lesion at the anterior hard palate contiguous with the nasal septum. A two-staged treatment was performed, starting with a minimal invasive access Brown class 2a maxillectomy guided by a patient-specific cutting guide. Pending histological confirmation, an obturator prosthesis was placed to seal the oroantral communication. Histopathology confirmed a low-grade chondrosarcoma with clear margins of at least 5 mm. A second-stage reconstruction was performed a year later using a posterior pedicle lateral nasal wall flap (inferior turbinate flap) and palatal rotation flap restored nasal lining and oral mucosa. This approach achieved oncologic clearance with excellent functional outcomes. The case highlights the value of image-guided maxillectomy and staged regional flap reconstruction.

## 1. Introduction

Chondrosarcoma of the head and neck region represents approximately 0.1% of all tumors in this area [1,2]. Within the maxillofacial skeleton, the maxilla and nasal septum may occur as primary sites; however, they are considered uncommon locations. Low-grade chondrosarcomas typically exhibit slow but locally invasive growth and often present as a painless swelling, which may lead to delayed diagnosis [1,3].

Histologically, chondrosarcomas are characterized by a lobulated growth pattern with abundant blue grey chondroid matrix. The tumor is composed of irregular cartilage lobules of varying size and shape, separated by fibrous septa and frequently permeating pre-existing bony trabeculae. Chondrosarcomas are histologically classified into four grades based on cellularity, nuclear atypia, mitotic activity, and growth pattern: grade I (low-grade/atypical cartilaginous tumor); grade II (intermediate-grade); grade III (high-grade), with increasing atypia, cellularity, and mitotic figures; and grade IV. Grade IV, or dedifferentiated chondrosarcoma, is characterized by the presence of a high-grade non-cartilaginous sarcomatous component and is associated with an aggressive clinical course and poor prognosis [4]. Differentiation from benign chondroma can be difficult, as both lesions share overlapping radiographic and histopathologic characteristics, particularly when cellular atypia is minimal [2,5]. Complete surgical excision with negative margins is the primary treatment [1,2,5,6,7].

Reconstruction of premaxillary defects can be achieved using a range of prosthetic and surgical options, including obturator prostheses, local and regional flaps, and microvascular free tissue transfer, with the choice guided by defect size, involvement of adjacent structures, and functional requirements. For large skull-base defects, the posterior pedicle lateral nasal wall flap has been described as a reliable reconstructive option, providing a well-vascularized nasal lining based on the sphenopalatine artery. The flap enables tension-free coverage of broad reconstructive areas while maintaining excellent tissue viability and fostering rapid mucosal healing. The consistent vascular anatomy of the flap ensures reliable perfusion, even in challenging surgical scenarios [8]. Multilayer closure of oronasal communication further reduces fistula formation, as demonstrated in a triple-layer endonasal flap technique [9].

## 2. Case Presentation

### 2.1. Patient History

**Figure 1:**
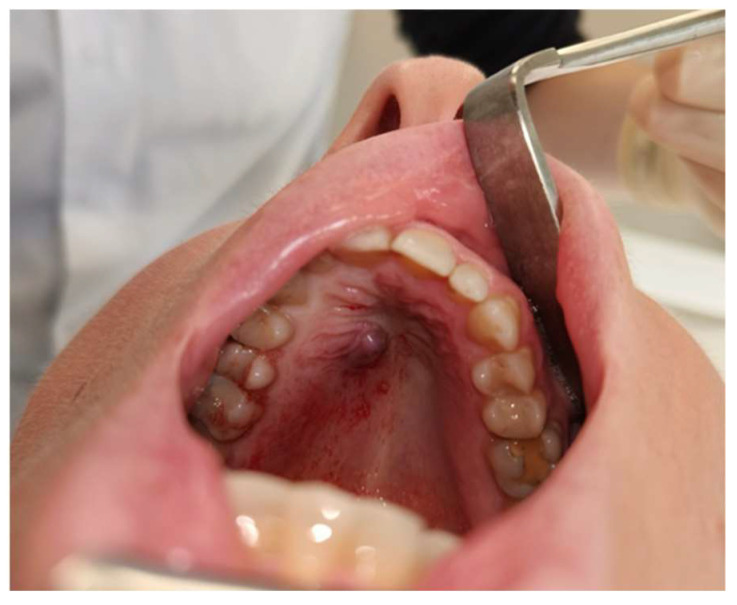
Clinical intraoral presentation of the recurrent palatal mass during consultation in January 2024.

A 62-year-old woman presented with a slowly enlarging, painless swelling on the anterior hard palate that she had noticed for approximately six months [Figure 1]. She reported mild sensitivity when consuming acidic foods but no pain, bleeding or weight loss. Medical history included hyperlipidemia treated with atorvastatin. She was a lifelong non-smoker and consumed moderate amounts of alcohol (7–10 units/week).

### 2.2. Previous Interventions

In January 2013, the patient underwent resection of a palatal lesion that was histologically inconclusive, with features insufficient to definitively distinguish between chondroma and chondrosarcoma. Postoperative magnetic resonance imaging (MRI) performed in September 2013 demonstrated residual disease. Following multidisciplinary team (MDT) discussion, close clinical surveillance with intermittent MRI follow-up was initiated. Imaging performed at four-year intervals remained stable until 2022, when the midline palatal mass recurred and increased in size. MRI demonstrated an ovoid, contrast-enhancing lesion at the anterior hard palate, contiguous with the nasal septum, and repeat resection revealed a benign chondroid tumor without arguments suggesting chondrosarcoma.

**Figure 2:**
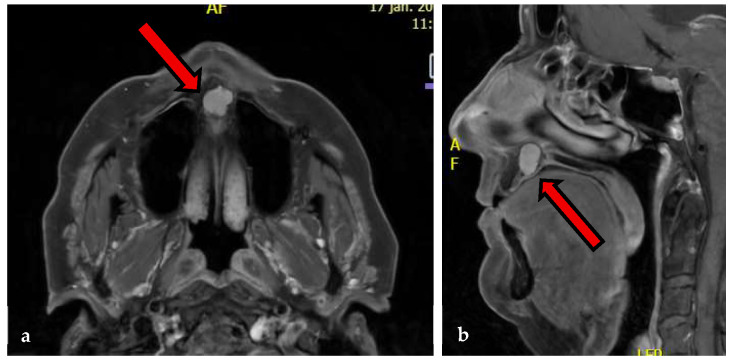
(**a**) Preoperative axial MRI with contrast showing an enhancing lesion (red arrow) at the anterior hard palate in close relation to the nasal septum. (**b**) Preoperative sagittal MRI showing lobulated contrast-enhancing lesion (red arrow) at the anterior hard palate with extension toward the nasal septum and maxillary crest.

**Figure 3:**
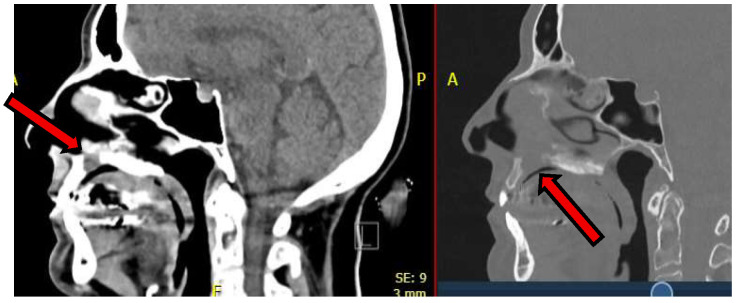
Sagittal computed tomography (CT) imaging demonstrating the anterior hard palate lesion (red arrow).

In February 2024, the patient returned with another recurrence. MRI revealed a lobulated lesion measuring 12 mm × 11.5 mm × 13 mm with intermediate T1 and hyperintense T2 signal and strong contrast enhancement, consistent with a recurrent chondroid tumor [Figure 2 and Figure 3]. No cervical lymphadenopathy was noted. Biopsy demonstrated increased cellularity of the chondroid tissue without mitoses or high-grade atypia, raising suspicion of a recurrent low-grade chondrosarcoma.

### 2.3. Surgical Management

Following multidisciplinary oncologic team discussion, a two-stage management plan was agreed upon.

#### 2.3.1. Stage 1: Tumor Resection and Immediate Obturator Placement

**Figure 4:**
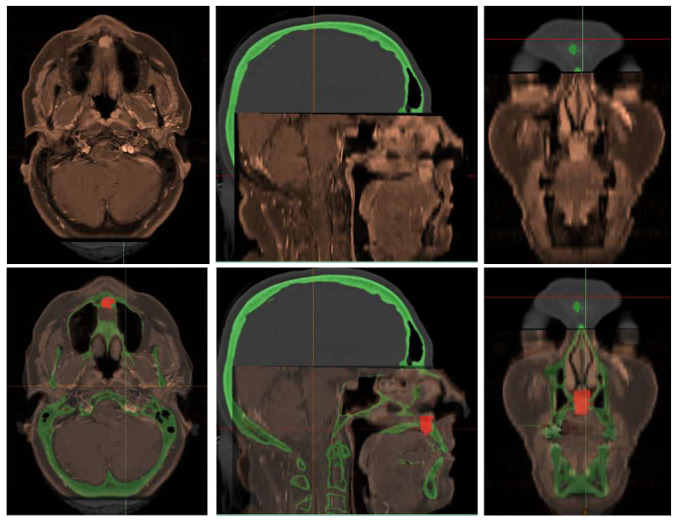
CT-MRI fusion images used for preoperative planning of a Brown class 2a guided maxillectomy. Registration of the MRI (orange) on to the CT scan (grey) was performed voxel based, using maxillary bony structures and airway. Virtual planning image showing tumor (red) and 3D bone model (green) used for fabrication of the patient-specific cutting guide. Materialise Mimics (Materialise NV, Leuven, Belgium) was used.

**Figure 5:**
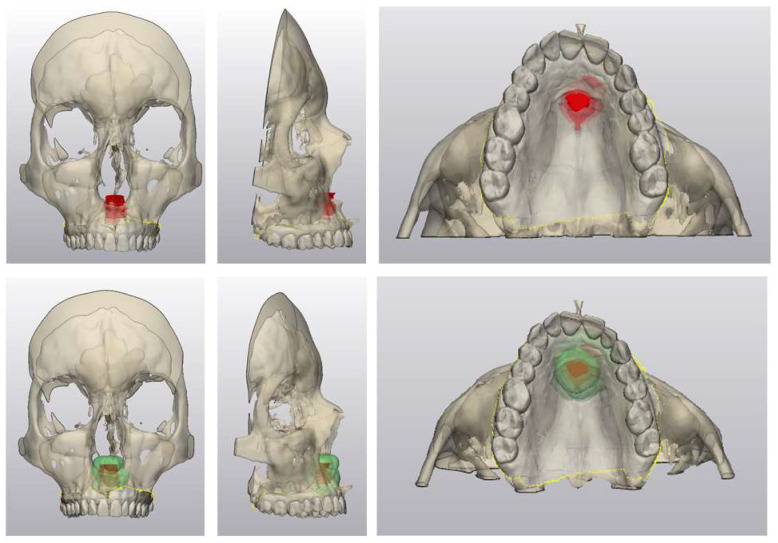
Virtual surgical planning based on CT–MRI fusion images, showing 3D segmentation of the tumor (red) and planned resection margins (green). Fusion of CT and MRI allows precise delineation of the palatal chondrosarcoma (red). The green volume represents the planned osteotomy margin that will be resected en bloc together with the tumor to achieve oncologic clearance. Patient-specific cutting guides were designed based on this model.

**Figure 6:**
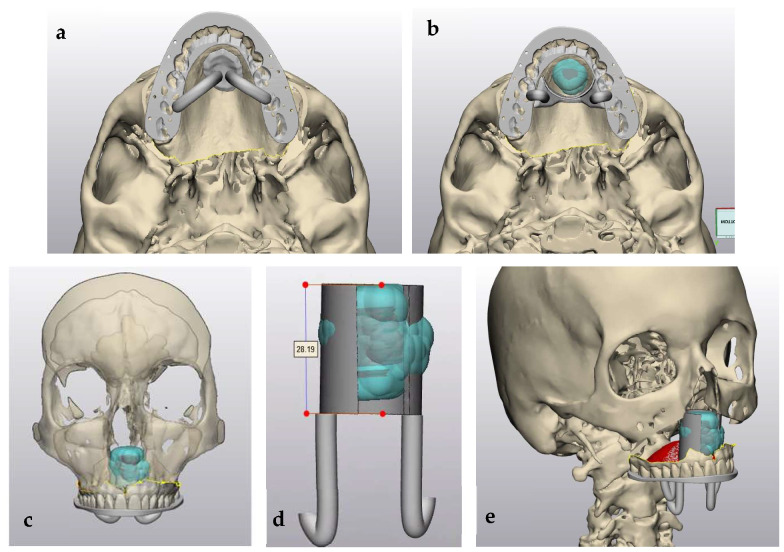
(**a**–**e**) Patient-specific access guide for palatal soft-tissue resection and sliding bone-cutting guide with cutting angle and tumor depth reference. The removable sliding tube allows controlled osteotomy depth using a 28 mm burr (red). The palatal chondrosarcoma with a planned 5-mm resection margin is shown in blue. (**a**) A patient-specific, mucosa-supported cutting guide for palatal soft-tissue resection. (**b**–**e**) Following initial soft-tissue resection and elevation of the palatal mucosa, a second patient-specific bone-cutting guide incorporating a sliding tube mechanism was applied.

**Figure 7:**
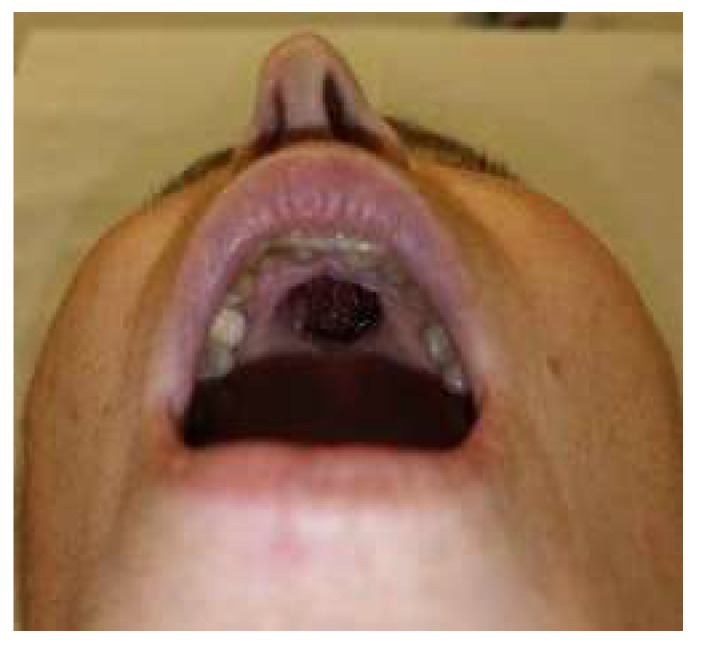
Intraoral view 14 days after stage 1 surgery showing the palatal defect, with oronasal communication.

**Figure 8:**
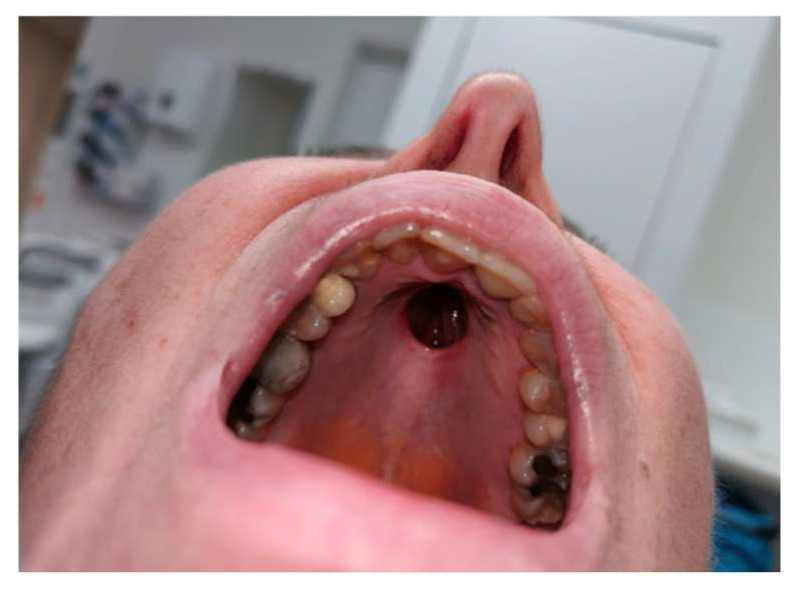
Intraoral view six months after stage 1 surgery showing a stable, epithelialized palatal defect with unobstructed communication to the nasal cavity.

A limited Brown class 2a maxillectomy with apicotomy of the anterior teeth was performed by a maxillofacial head and neck surgeon to ensure adequate margins while preserving function. Based on the CT-MRI fusion images, an in-house-printed patient-specific surgical guide was dental supported by fusing this to a wafer. One guide was designed for the soft tissue mucosal resection and a second one to guide the extent and angle of the osteotomies with the help of a sliding tube supported on the palatal base, enabling precise tumor resection with a planned 5 mm safety margin [Figure 4, Figure 5 and Figure 6]. The lesion was excised en bloc. An immediate obturator prosthesis was inserted to restore palatal continuity and facilitate speech and swallowing. The postoperative evolution of the palatal defect after tumor resection is shown in Figure 7 and Figure 8.

##### Segmentation and Guide Planning

Preoperative virtual planning was performed in-house by the maxillofacial surgeon using fused computed tomography (CT) and contrast-enhanced MRI datasets to accurately delineate the tumor extent and define resection margins. MRI–CT fusion was carried out using Materialise Mimics 17 to depict the tumor contours, after which a circumferential 5 mm safety margin was applied to generate the planned resection volume [Figure 4 and Figure 5].

Based on this model, a tooth-supported positioning splint was designed by matching intraoral scan data with the CT images, ensuring accurate and reproducible intraoperative guide placement using Materialise Enlight (version 6.1). Subsequently, a patient-specific, mucosa-supported cutting guide for palatal soft-tissue resection was designed separately, as soft-tissue resection was more limited than the planned bony resection [Figure 6a].

Following initial soft-tissue resection and elevation of the palatal mucosa, a second patient-specific bone-cutting guide incorporating a sliding tube mechanism was applied. Both the soft-tissue cutting guide and the bony sliding tube guide were designed using Materialise 3-matic version 18. The sliding tube allowed controlled drilling along the planned osteotomy trajectory, providing accurate control of both resection angle and depth and minimizing the risk of unnecessary lateral extension into the nasal cavity or septal cartilage [Figure 6b–e].

The superior osteotomy margin was completed via a limited Le Fort I approach at the level of the superior tip of the sliding tube, enabling precise sectioning at the planned superior border [Figure 6d,e]. The guides were printed in-house by the maxillofacial surgeon on a Stratasys MediJet SLA-printer (Stratasys Ltd., Rehovot, Israel and Eden Prairie, MN, USA) in biocompatible MED615 material that has been undergoing a sterilization process according MDR regulations.

#### 2.3.2. Histopathologic Findings

Microscopic examination of the resection specimen showed a chondroid tumor with increased cellularity, mild chondrocyte atypia with occasional bi- and multinucleated cells, transition into fibrous connective tissue and no evidence of bone entrapment or encasement. All resection margins were free of tumor. The tumor was completely excised with a minimum margin of 5 mm and had a maximum diameter of 1.6 cm. The additional incisional specimen from the septum contained normal, non-atypical cartilage with no evidence of residual tumor. Immunohistochemistry demonstrated strong S100 expression, confirming chondrogenic differentiation, and a mildly elevated proliferative index (Ki-67).

Despite morphological uncertainty between chondroma and chondrosarcoma, the diagnosis was based on an integrated evaluation of clinical, radiological and histopathological findings. The recurrent disease at the same anatomical site, lobulated contrast-enhancing growth on MRI, increased cellularity with mild atypia, fibro-chondroid matrix transition and mildly elevated proliferative index favored a diagnosis of grade 1 chondrosarcoma over benign chondroma. These findings are consistent with established diagnostic criteria in which high cellularity, nuclear changes (e.g., binucleation, open chromatin) and matrix alterations (e.g., fibrous transition) are considered key features distinguishing low-grade chondrosarcoma from chondroma. Patient age and recurrent behavior further supported classification as a low-grade chondrosarcoma [10].

#### 2.3.3. Stage 2: Definitive Reconstruction

**Figure 9:**
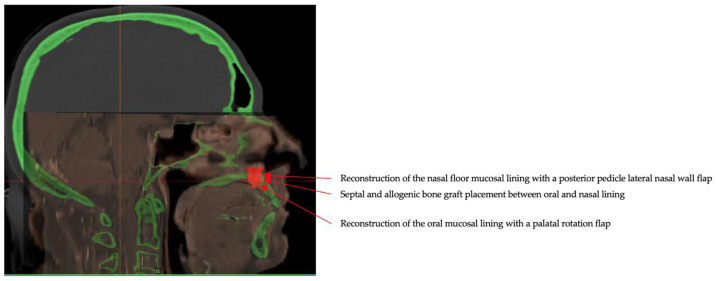
Sagittal CT-MRI fusion image illustrating multilayer septal–palatal reconstruction, including reconstruction of the nasal floor mucosal lining with a posterior pedicle lateral nasal wall flap, interposition of a septal and allogenic bone graft between the nasal and oral linings and reconstruction of the oral mucosal lining using a palatal rotation flap.

**Figure 10:**
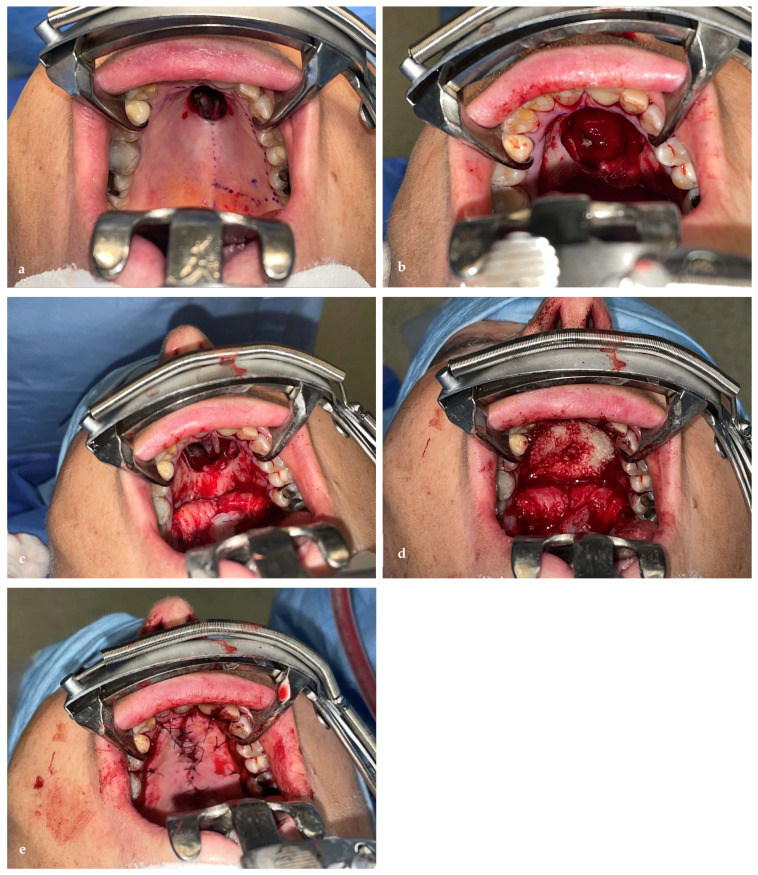
Intraoperative images of stage 2 reconstruction. (**a**) Marking of the mucosal incisions on the hard palate to ensure clear visualization of the defect. (**b**,**c**) Exposure of the defect in the hard palate. (**d**) Following repair of the nasal lining with the posterior pedicle lateral nasal wall flap, bone graft placement was performed. (**e**) Final result of the palatal rotation flap.

**Figure 11:**
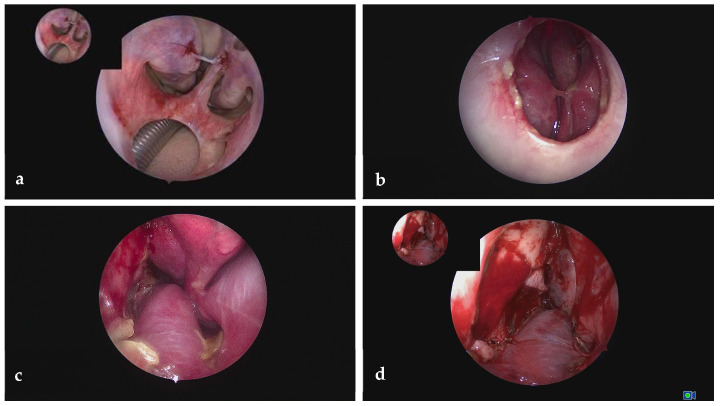
Endoscopic images during reconstruction. (**a**) Preoperative intranasal view of the palatal defect. (**b**) Preoperative intraoral view of the palatal defect. (**c**) Intraoperative image of the posterior pedicle lateral nasal wall flap. (**d**) Postoperative image of the rotated posterior pedicle lateral nasal wall flap draped over the palatal defect creating a new nasal layer.

After histologic confirmation of free margins, definitive reconstruction was scheduled one year later. A lateral nasal wall and inferior turbinate flap with a posterior pedicle was used to reconstruct the nasal floor mucosal lining by the ENT surgeon, combined with a septal and allogenic bone graft and a palatal rotation flap to close the oral side of the defect, which was then performed by the Maxillofacial head and neck surgeon [Figure 9, Figure 10 and Figure 11].

Harvesting of the posterior pedicle lateral nasal wall flap begins with making incisions via an endonasal endoscopic approach using unipolar electrocautery with an extended insulated needle tip. An anterior incision is made along the anterior edge of the ascending part of the frontal process of the maxilla, extending inferiorly toward the head of the inferior turbinate. A second incision runs vertically along the posterior aspect of the lacrimal bone, extending inferiorly to a level near the upper border of the inferior turbinate. A third incision is made to connect the superior ends of the first two incisions. The anterior incision can be extended downward along the lateral nasal wall, just anterior to the inferior turbinate, and continued across the nasal floor, allowing for the harvest of a larger flap if necessary. At its most inferomedial point, this incision meets another cut oriented in the sagittal plane that runs anteroposteriorly until reaching the level of the sphenopalatine foramen. Thus, this anteroposterior incision runs either along the lateral nasal wall or extends across the nasal floor to obtain a larger flap. The incision over the lacrimal bone merges with a sagittal incision running anteroposteriorly over the upper border of the inferior turbinate, terminating at the sphenopalatine foramen. A horizontal incision along the nasal floor, oriented in the coronal plane, is made to connect the superior and inferior sagittal incisions at the level of the sphenopalatine foramen. The mucoperiosteum is then carefully elevated using a blunt instrument. Special attention must be given to the posterior portion of the flap, where the branches of the sphenopalatine artery must be identified and preserved. A fully elevated flap can be rotated, using the sphenopalatine foramen as its pivot point.

Restoration of the intermediate osseous layer was achieved using a septal cartilage graft, which was reinforced with allogenic cadaveric cancellous bone graft. The graft components were stabilized using a fibrin sealant (Tisseel, Baxter), providing structural support and facilitating integration.

Closure of the oral side of the defect was completed using a palatal rotation flap. A circumferential mucosal margin of approximately 5 mm was preserved around the adjacent teeth to reduce the risk of wound dehiscence. Careful dissection was performed around the greater palatine neurovascular bundle to maintain flap vascularity and ensure reliable rotation without tension.

This three-layer reconstruction, comprising vascularized nasal lining, rigid intermediate structural support, and well-vascularized oral mucosal coverage, provided a robust barrier and minimized the risk of persistent oronasal fistula formation [Figure 10 and Figure 11].

### 2.4. Postoperative Course and Follow-Up

**Figure 12:**
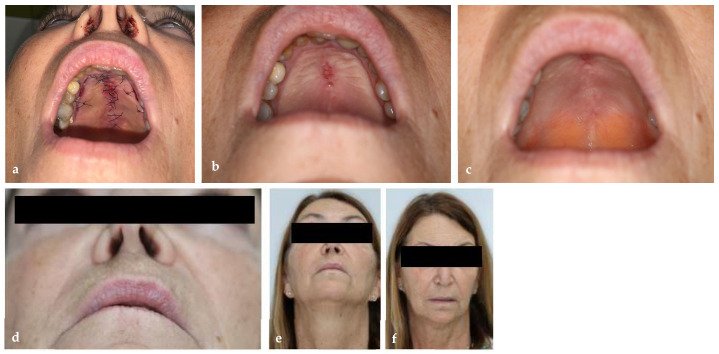
Postoperative healing and esthetic outcomes after stage 2 reconstruction. (**a**) Postoperative day 7. (**b**) Postoperative day 14. (**c**) Postoperative day 21. (**d**–**f**) Extraoral outcomes.

The early postoperative course was uncomplicated. Mucosal healing was complete without infection or dehiscence [Figure 12]. The obturator restored intelligible speech and normal diet. MRI at three months revealed mild right-sided enhancement interpreted as postoperative fibrosis rather than recurrence. Subsequent MRI at one year after surgery showed no signs of recurrence or residual disease, hence allowing us to proceed with second-stage reconstruction. Clinically, the palatal surface was smooth and intact.

**Figure 13:**
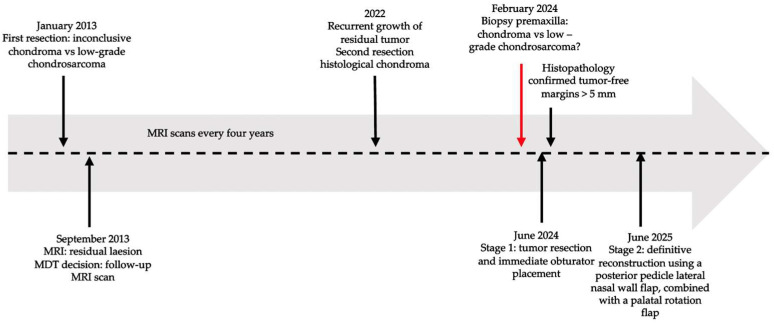
Chronological timeline of the patient’s clinical course with a two-stage surgical approach. The red arrow marks the moment when the patient was first managed at our department.

Following reconstruction in June 2025, both posterior pedicle lateral nasal wall and palatal flaps healed uneventfully. At three-week follow-up, there was complete mucosal coverage. Six months after reconstruction, the patient reported normal speech and swallowing and MRI showed no evidence of residual or recurrent tumor. Figure 13 illustrates the chronological timeline of the patient’s clinical course.

## 3. Discussion

### 3.1. Minimally Invasive Resection Strategy

In anterior maxillary tumors, the objective is to obtain oncologic clearance while preserving speech and swallowing. Conventionally, anterior maxillary and premaxillary tumors are approached through an open Le Fort I down-fracture, providing wide exposure of the anterior palate, nasal floor and maxillary alveolus. Achieving adequate deep and peripheral margins often necessitates removal of the anterior maxillary dentition, including the central and lateral incisors due to their proximity to the tumor and the limited visibility and access afforded by transoral routes. As a result, traditional approaches may lead to significant defects with functional and esthetic consequences, including anterior tooth loss and alteration of the nasolabial support. This case is unique due to the use of a minimally invasive approach for the management of low-grade chondrosarcoma of the hard palate, specifically utilizing a patient-specific cutting guide for tumor resection, allowing preservation of the anterior teeth and nasal base support. CT–MRI fusion allowed precise delineation of tumor dimensions and defined the relationship to adjacent structures and the depth toward the nasal cavity. Based on these fused datasets, a patient-specific sliding bone-cutting guide was developed. This design incorporated: a cutting-angle reference, a tumor-depth indicator and a removable sliding tube constraining drill trajectory. Wong et al. demonstrate that integrating anatomical and functional data for 3D surgical planning in bone tumors is technically feasible, enabling reliable and potentially beneficial resections with navigation assistance [11].

To our knowledge, no reports have been published describing the use of a patient-specific cutting guide in the surgical management of a chondrosarcoma of the hard palate. However, comparable approaches have been documented for other anatomical locations and tumor types. Several studies have demonstrated the feasibility of 3D-printed, computer-aided design/computer-aided manufacturing (CAD/CAM)-based osteotomy guides for pelvic, mandibular and maxillary resections, as well as for orthognathic procedures. These guides are primarily designed to control the osteotomy orientation and margins, thereby improving surgical precision and facilitating accurate reconstruction [12,13,14]. Although the principle is transferable to the premaxillary region, no published case has yet combined angle and depth control in a cutting guide specifically applied to chondrosarcoma of the hard palate. The technique thus minimizes bone sacrifice and tissue disruption while maintaining oncologic reliability.

### 3.2. Rationale for Delayed Reconstruction and One-Year Interval

A wide surgical excision of chondrosarcoma is described, including the hard palate and maxilla, followed by either immediate or short-interval reconstruction, typically within weeks or months of the initial surgery. The rationale for immediate or early reconstruction includes improved quality of life in the short term, fast restoration of speech and swallowing and better outcomes in mastication and phonation [15,16].

A one-year interval between tumor resection and reconstruction was chosen to allow mucosal regeneration and to monitor for potential residual disease before performing complex reconstruction. Early reconstruction, often performed within weeks of resection, may compromise oncologic surveillance and carries the risk of flap failure if secondary resection becomes necessary. Delayed reconstruction permits confirmation of local control and ensures a well-vascularized and epithelialized bed suitable for reliable flap integration.

### 3.3. Staged Multilayer Reconstruction Compared with Other Techniques

Compared with traditional techniques, the presented approach differs substantially in both timing and invasiveness. Classic maxillectomy with immediate reconstruction typically involves wide surgical resections followed by immediate soft-tissue or bone flap reconstruction. While this provides rapid anatomical closure, it often results in greater physical and esthetic morbidity and allows limited time for mucosal healing. Reconstruction after anterior palatal tumor resection generally ranges from prosthetic obturation to flap reconstruction. A systematic review comparing obturators with surgical flap reconstruction found largely similar global functional outcomes, except for improved mastication efficiency, speech intelligibility and lower oral pain scores in the flap group [15]. Obturators can restore speech and swallowing quickly but are less effective for large or combined defects, particularly when nasal lining is missing. Free or rotational flaps tend to yield better long-term outcomes in mastication and phonation but require more extensive surgery and may hinder early detection of recurrence [15,16]. These findings support a staged management strategy consisting of immediate obturator placement to maintain speech and swallowing during postoperative healing, followed by delayed flap reconstruction.

A delayed, minimally invasive approach was adopted in this case. After one year, a multilayer reconstruction was performed, restoring both nasal and oral linings with interposed bone grafts from septal bone and an allogenic bone graft. This layered reconstruction principle, described by Badaoui et al., emphasizes independent restoration of the nasal lining, skeletal framework and oral lining to minimize oronasal fistula formation [9]. In contemporary case reports, immediate reconstruction is often achieved using free flaps such as the radial forearm (RFFF) or anterolateral thigh flap. Although these single-stage reconstructions provide immediate separation of the oral and nasal cavities, they are more invasive. RFFF can cause severe morbidity at the donor site, mainly due to exposure of the flexor tendon that results from a failed harvesting of the skin graft, changed sensation in the radial nerve, esthetic deformity and reduced grip strength [17]. The combination of a posterior pedicle lateral nasal wall flap for nasal lining, palatal rotation flap for oral lining and interposed bone graft recreated functional partitioning with high vascular reliability. This staged method balances oncologic prudence with functional and esthetic restoration while avoiding the morbidity of immediate free-flap reconstruction.

The inferior turbinate has a dual blood supply: posteriorly from the posterolateral nasal artery (a branch of the sphenopalatine artery) and anteriorly from the branches of the superior labial and anterior ethmoidal arteries. This vascular pattern allows its use as either an anteriorly or posteriorly based pedicled flap. The use of an anterior pedicle lateral nasal wall flap has been described for the reconstruction of residual palatal fistulas following cleft palate repair [18]. For skull-base defects, reconstruction using a posterior pedicle lateral nasal wall flap has been reported [8,19]. Further, Darr et al. described a three-layered closure technique combining the buccal fat pad, a buccal mucosal advancement flap and a posterior pedicle lateral nasal wall flap for the repair of oroantral fistulas [20]. However, there are no published reports describing the use of a posterior pedicle lateral nasal wall flap for the reconstruction of an anterior palatal defect following tumor resection of a chondrosarcoma.

When the soft palate is substantially involved, combining a radial forearm free flap with a superiorly based pharyngeal flap further improves speech and swallowing outcomes [21]. Medium-sized palatal defects and postoperative oronasal fistulae may be closed using facial artery musculomucosal flaps, which offer an axial blood supply, low donor-site morbidity and favorable functional recovery, although they do not restore nasal mucosa [22,23].

### 3.4. Prognosis and Follow-Up

Five-year overall survival rates for low-grade (grade I) chondrosarcoma in the head and neck region ranges from 70% to 90% [1,2,24,25]. There is a 10% risk of metastasis for grade 1 chondrosarcoma [25]. The completeness of oncologic resection is the most critical prognostic factor, as incomplete excision or positive margins markedly increase the likelihood of local recurrence. Recurrence rates of approximately 15–17% have been reported within five years, but this can be higher if margins are suboptimal and in higher grades of chondrosarcoma [20]. Most recurrences occur locally and within the first few years after treatment, but late recurrences (sometimes more than a decade after treatment) have been documented. Imaging-based surveillance, preferably with MRI, should be performed at regular intervals [6]. Radiotherapy is generally reserved for unresectable or high-grade lesions, as low-grade variants show limited radiosensitivity [1,3,5].

### 3.5. Limitations

This case report describes a single patient, which limits the generalizability of the findings. Follow-up time remains limited and longer-term surveillance is required to confirm sustained oncologic control. Additionally, the patient-specific cutting-guide workflow requires advanced imaging, digital planning and technical expertise, which may not be available in all clinical settings and may limit broader implementation.

## 4. Conclusions

We report a case of recurrent low-grade chondrosarcoma of the hard palate successfully managed through image-guided limited resection and staged septal–palatal reconstruction. The use of a patient-specific cutting guide enabled precise tumor removal with preservation of critical structures and maintenance of function. Restoration of nasal lining with a posterior pedicle lateral nasal wall flap, combined with a palatal rotation flap, provided durable separation of the oral and nasal cavities and prevented fistula formation. This two-staged reconstruction approach offers a balance between oncologic safety and quality of life. Early biopsy, long-term surveillance and multidisciplinary planning are essential for optimal outcomes in recurrent cartilaginous tumors of the palate.

## Data Availability

The data supporting the findings of this study are not publicly available due to the privacy and confidentiality of participant responses. An anonymized version of the dataset may be made available from the corresponding author upon reasonable request.

## Abbreviations

The following abbreviations are used in this manuscript:

MRI	Magnetic resonance imaging
MDT	Multidisciplinary team discussion
CT	Computed tomography
CAD	Computer-aided design
CAM	Computer-aided manufacturing
RFFF	Radial free forearm flap
